# Individual differences in children's innovative problem-solving are not predicted by divergent thinking or executive functions

**DOI:** 10.1098/rstb.2015.0190

**Published:** 2016-03-19

**Authors:** Sarah R. Beck, Clare Williams, Nicola Cutting, Ian A. Apperly, Jackie Chappell

**Affiliations:** 1 School of Psychology, University of Birmingham, Birmingham, West Midlands, B15 2TT, UK; 2 School of Biosciences, University of Birmingham, Birmingham, West Midlands, B15 2TT, UK

**Keywords:** innovation, cognitive development, individual differences

## Abstract

Recent studies of children's tool innovation have revealed that there is variation in children's success in middle-childhood. In two individual differences studies, we sought to identify personal characteristics that might predict success on an innovation task. In Study 1, we found that although measures of divergent thinking were related to each other they did not predict innovation success. In Study 2, we measured executive functioning including: inhibition, working memory, attentional flexibility and ill-structured problem-solving. None of these measures predicted innovation, but, innovation was predicted by children's performance on a receptive vocabulary scale that may function as a proxy for general intelligence. We did not find evidence that children's innovation was predicted by specific personal characteristics.

## 1. Introduction

Cultural modification is a two-stage process. First, a novel idea must emerge (innovation). Second, it must be transmitted through the group (cultural transmission). In this paper, we are concerned with the question of how novel ideas arise in individuals and specifically whether some individuals are more likely to come up with innovations than others. Although some innovations will come about by chance or uninformed trial and error, this paper will focus on the cognitive processes that may underpin purposeful attempts at innovative problem-solving.

One possibility is that there are unusual individuals who solve problems beyond the reach of the vast majority of their peers. For example, research with non-human species often describes behaviour by one individual in a population: Betty the crow, who made a hook tool to fish a bucket from a tall tube [1]; Imo, the macaque, who washed sweet potatoes [2]; Mike, the chimpanzee, who banged cans together to make a threatening display [3]. Similarly, we can identify a few humans who are thought of as particularly prolific or radical innovators, such as Steve Jobs or Thomas Edison. Given the rarity both of individuals and instances of innovative behaviour, it is difficult to infer the general characteristics of innovators from these unusual cases. Furthermore, the characteristics of these unusual innovators may not be the same characteristics as underpin less dramatic innovations in the general population of a species.

Thus, we can also look for innovation ability across more typical members of a population. As the tendency to innovate seems to vary within groups, we can try to identify the characteristics that promote this behaviour. This approach leads researchers to look across groups of individuals, present them with innovation challenges, and hope to identify characteristics of those who innovate. Laland and Reader adopted this approach in their studies of guppies. Individual guppies who have innovated once are more likely than others to do so again [4]. Laland and Reader identify two levels at which these characteristics are found: state-dependent factors and personality traits.

### (a) Individual differences

State-dependent factors^1^ identify classes of individuals within a population and include characteristics such as sex and age. In guppies, innovators are more likely to be female than male. Laland and Reader attribute this finding to the fact that females have unconstrained growth (unlike males who stop growing at maturity) and are larger, arguing that female fish are more likely to benefit from innovations when foraging (see discussion [5]). Evidence from non-human primates suggests that age, another state-dependent factor, may influence innovation: there are some reports of more innovations by adults [6], but others identify a high number of innovations in youngsters (e.g. [7]). Social status may also be important: in chimpanzees subordinate individuals are more likely to be recorded as innovators than dominant [6]. In recent studies with humans where individuals had to innovate solutions to simple physical problems, older children perform better than younger [8,9] and adults typically solve these tasks with ease [8].

Personality traits are also potential explanations for individual differences in innovative behaviour. The biologists' use of this term goes beyond the dispositions that psychologists might traditionally think of as *personality* by including cognitive abilities and motivational tendencies. Here, we will call them personal characteristics to bridge the fields [10]. Some personal characteristics have been identified as predictors of innovation success. For example, spotted hyenas who show more diverse initial exploratory behaviour and less neophobia are more likely to innovate and solve a problem [11]. The same characteristics also predict innovation in Carib grackles (*Quiscalus lugubris*, a New World member of the passerine family [12]). In this study, the personal characteristics measures were not taken in the problem-solving task used to measure innovation. For example, a neophobia score was given by the length of time it took a bird to start feeding after a novel object had been introduced into the cage. Finding relationships between innovation and measures taken out of this context adds weight to the idea that general personal characteristics are involved in innovation.

Research with human adults offers some suggestions for personal characteristics that may be associated with innovation. Social psychological studies (e.g. [13]) have examined the characteristics associated with particular groups of creative people (e.g. artists versus non-artists) and found that the more creative samples scored more highly on characteristics such as openness and impulsivity among others (these two characteristics are particularly interesting because of the resonance with the non-human literature). Simonton [10] emphasizes that personal characteristics such as non-conformity and risk taking (along with openness and others) interact with cognitive processes, lifespan development (an individual's experiences throughout their life) and social context to produce innovators.

Recent work that we describe in more detail below shows that children's ability to innovate solutions to problems improves with age. However, another state-dependent factor, gender, does not differentiate performance in any of these studies. With regard to personal characteristics, we have yet to explore whether these predict children's innovative ability. This is the focus of this paper. Outside the realm of innovation, there are several domains in which children's personal characteristics seem to explain their abilities. For example, one can predict which children will pass a false belief task based on their performance on inhibitory control measures [14]. Individual differences studies have also suggested that in order for children to experience regret (something that emerges around 6 or 7 years) children need to develop advanced attentional flexibility powers [15]. Recent work indicates that children with better working memory are better liars (indicating more advanced cognitive ability [16]). Investigating individual differences during childhood reminds us that the skills needed to acquire and deploy abilities as a novice may well be different from those required by the mature system. For example, while language seems to play a critical role in children's acquisition of theory of mind abilities, severe damage to language abilities in adulthood leaves the same theory of mind reasoning apparently intact [17]. The personal characteristics that may explain the emerging ability to innovate in young children may be different from those that underpin innovation in adults.

### (b) Problem-solving studies

To identify personal characteristics that underpin innovation ability in children, we will exploit recent findings that identified children's surprising difficulty with innovation in physical problem-solving tasks. These studies are based on a task from the non-human literature in which birds made novel hook tools to retrieve an otherwise unreachable item (see [1,18,19]).

In the child version of the task, participants are presented with a tall, transparent tube at the bottom of which is a bucket containing a desirable sticker. Various materials are offered to help the child retrieve the bucket, some of which can be used to fashion a hook tool. In most studies, children have been given pliable pipecleaners to bend [8], although sometimes pieces of wood have been used [20]. Children find the hook-innovation task rather difficult to solve. Three- to four-year-olds almost all fail, and it is not until around the age of 8 years that the majority of children succeed in innovating a suitable tool. Between 5 and 8 years of age around half of the children solve the task [8].

Subsequent studies have confirmed the finding and ruled out some alternative explanations for children's difficulties, suggesting that the tool-making paradigm is an interesting one to investigate the development of innovation. Children's difficulties are not simply the result of a pragmatic misunderstanding that the materials should not be manipulated: performance remained poor when they were directed to ‘make something’ with the materials [21] and after they had the chance to manipulate the materials in a familiarization phase [8]. This latter evidence also shows that children fail the task even when they have just seen that pipecleaners are pliable. Children appear to know that the hook tool is a suitable solution to the problem: if given a choice between a straight pipecleaner and a premade hook children as young as 4 choose the hook to retrieve the bucket, and if children are shown how to make (but not use) a hook by a demonstrator they rapidly make their own tool and spontaneously use it successfully [8].

The studies described, thus far, have been conducted with children from one WEIRD cultural group [22]. However, Neilsen *et al*. [23] found very similar levels of (lack of) success in 3–5-year-old South African Bushman children (and urban Australians). The Bushman children were of particular interest because in their communities there is ‘a lack of reliance on direct instruction in learning and greater exposure to needing to make artifacts that are played with’ (p. 386). Despite this difference in environment and experience, the Bushman children's performance was no different from the urban Australians', and indeed, none of the 24 Bushman participants innovated a hook tool from a straight pipecleaner.

Furthermore, children's difficulties with innovation are not limited to the bucket retrieval/hook task—they extend to other problems. Children struggle (although to a lesser extent) to unbend a pipecleaner making a long straight tool that can push a ball from a horizontal tube [21]. They also have difficulty with tasks that involve flattening a sheet of pliable material and (separately) removing a stick from a piece of cardboard in each case to make a ‘shelf’ that helped to solve a puzzle [20]. Tennie *et al*. [24] gave children the task of pulling an out-of-reach platform towards them. The platform had a screw protruding from it and the solution was to bend a thin strip of pliable wood (‘wooden wool’) and loop it over the screw. None of the 4-year-olds tested spontaneously produced a loop. Hanus *et al.* [9] gave children a different innovation problem in which they could use water to float a peanut to the top of a tube. Testing children across a range of ages from 4 to 8 years, the levels of success reported are remarkably similar to those seen on the hook-making task: only 9% of 4-year-olds retrieved the peanut, whereas 42% of 6-year-olds and 58% of 8-year-olds solved the task. It is notable that in the middle-childhood age range (6- and 8-year-olds) around half of the children solved the task. As in the hook-making task, there was substantial individual variation in performance within these age groups. A similar lack of innovative use of the water as a tool by 4-year-olds was reported by Nielsen [25].

We, and others including [26,27], have tended to interpret these findings as showing simply that tool innovation is challenging for young children and that majority success emerges remarkably late (around 8 years old). However, this does not truly exploit the developmental trajectory in the data. We have most evidence from the hook-innovation task and on closer inspection we see that between the phases of near floor performance in the under 5 s and near ceiling performance in the over 8 s, there is an extended period where performance is very variable. In the published samples of 5–8-year-olds, levels of success typically range around a third and a half of children succeeding. In the original Beck *et al*. study ([8], Experiment 2), it is particularly striking that there is an extended plateau of partial success by three age groups: 5–6, 6–7 and 7–8 year olds. Who are the children who pass and who fail? In this paper, we attempt to address this and present two individual differences studies in which we compared children's performance on the hook-making task to other personal characteristics that show variation in young children, in the hope of identifying the characteristics possessed by successful innovators.

## 2. Study 1: divergent thinking

In order to solve the hook-innovation task, one might imagine that children are required to think creatively and flexibly about the materials available to them and how to transform these into a solution. Divergent thinking describes the thinking process of generating multiple ideas and solutions. This is typically contrasted with convergent thinking, whereby a single correct solution is focused upon [28]. We speculated that, in order to generate an innovative solution to the hook task, children needed to think divergently about the materials available. Thus, we compared children's performance on the innovation task with measures of divergent thinking suitable for this age group.

In his structure of intellect model, Guilford [28] implicated fluency, flexibility and originality in divergent thinking. Similarly, Torrance [29] believed that those capable of producing lots of ideas during fluency tasks were more likely to think creatively than those who produce fewer ideas. In fluency tasks an individual has to come up with multiple possible ideas on a topic. For example, in the FAS, a verbal fluency task, participants are required to produce as many words as possible, beginning with one of the letters F, A or S, within a specified time [30]. We predicted that children who scored highly on measures of fluency, and thus could generate larger numbers of ideas and solutions, would also be more likely to perform well on tool-innovation tasks.

We presented children with two fluency tasks to measure divergent thinking. The first was similar to a measure used in the Torrance Tests of Creative Thinking [31] and involved children drawing as many different pictures as they could each based on a circle template. The second measure was used by Defeyter *et al.* [32] in their studies of functional fluency. Children have to suggest multiple uses for objects. This second fluency task involved thinking about objects and we were interested to see whether this might reveal a domain-specific relation with the tool-innovation task: i.e. that being able to think flexibly about objects would result in high levels of performance on both the object fluency task and the hook-innovation task. A second hypothesis was that performance on the object fluency task would be more strongly related to the hook-innovation task than the circles task.

## 3. Method

### (a) Participants

The final sample consisted of 40 participants aged 5–7 years (20 boys), mean age 6 years 5 months (6;5) (range 5;5–7;4) from a primary school in the West Midlands, UK. One further child was tested but excluded from analysis because he had overseen another child complete the hook-making task.

### (b) Procedure

Children were tested individually in one session by two female experimenters (CW and an assistant). The session lasted around 15–20 min and took place in a quiet space outside their school classroom. Children completed a short battery of tasks in a fixed order: circles task, hooks task, object uses task.

### (c) Circles task

Children were presented with a 21 cm × 30 cm piece of paper with a series of blank circles on it and a pencil. Two of the circles at the top of the page had already been used to draw a pig and a clock. Children were told ‘See these circles [points]. You can use them to make different drawings’. Their attention was then directed to the pre-drawn pictures and they were asked ‘Can you make as many different drawings as you can?’ Children were given 2 min to complete the task. As a child began drawing on a new circle, they were asked ‘What is this picture going to be?’ and the answer was noted by the experimenter. Neutral prompts were given by the experimenter where a child ceased to draw for 10 s or more, e.g. ‘Can you do any more drawings?’ After 2 min, children were praised and given a sticker and progressed to the next task.

Coding: Participants received a score for the total number of pictures drawn (excluding any duplications/replications). Categories of pictures were identified by two of the authors by reviewing the all the items drawn and identifying where there were common categories (i.e. where there were many similar pictures). This process was subjective (but see §4 for the similarity between analyses by items and by categories). These categories were: animals, people (where a whole person was drawn, including body), faces (including ‘face’, ‘head’ and other people, e.g. ‘me’ where only the face was drawn) and balls (e.g. ‘football’, ‘beach ball’). Any object drawn that did not fall into these categories was classed as being in a unique category of its own e.g. ‘wheel’, ‘pizza’. Pictures were then assigned to categories by one author (C.W.) and another coder (not blind). Agreement was 100%.

### (d) Hook-innovation task

Children were presented with the hook-innovation task based on Beck *et al*. [8] (Experiment 3). We included a familiarization phase in which the experimenter and child bent pipecleaners around a pen, in order to highlight the physical properties of the pipecleaner. In the main task, the experimenter showed the child the bucket containing a sticker at the bottom of the tall transparent tube and said that if s/he could get the sticker, s/he could keep it. A sole pipecleaner was made available to the child. The experimenter gave neutral prompts to encourage the child to attempt the task. If a child failed to solve the task after a minute, then the experimenter showed him/her how to make a hook (tool manufacture demonstration), after which the child had a further chance to retrieve the bucket.

*Coding*. Children were classed as passing or failing the hook-innovation task based on whether they retrieved the bucket using a hook in the first minute of the task.

### (e) Object uses task

Children completed a ‘functional fluency task’ based on Defeyter *et al*. [32]. Children were presented with two trials in a fixed order: first brick, then blanket. On each trial, children were shown the picture of the object, told its name and asked to generate as many different uses for the object as they could. The experimenter said ‘See this brick/blanket (pointed to picture). Think about the different things you can do with it. Tell me as many different things as you can’. Children were given a minute to list as many uses as possible. Then the picture was removed and they proceeded to the next trial.

*Coding*. One author (C.W.) and a blind-coder coded children's responses as suggestions (i.e. things that could be done with the object, including design and novel functions. See the electronic supplementary material). Comments that were merely descriptive and did not involve acting upon the object, e.g. ‘it is heavy’, ‘it is soft’ were excluded from analysis. Inter-coder agreement was 80% and disagreements were resolved by a third person. N.B. Unlike Defeyter *et al.* [32], we included suggestions that did not have a clear goal in our total because we were interested in divergent thinking, rather than functional fixedness.

Suggestions were then grouped into categories. Categories were identified (by C.W. and blind-coder independently) based on the children's responses and identifying where there were common categories (i.e. where suggestions shared a theme). This process was subjective (but see §4 for the similarity between analyses by items and by categories). For example, any suggested use of a brick for building/construction purposes, e.g. ‘build a tower’, ‘make a factory’, was assigned to the ‘building’ category. Any item not fitting into the defined categories was treated as belonging to a unique category of its own. Inter-rater agreement was good (94% for blanket categories and 100% for brick categories). Disagreements were resolved by a third person.

## 4. Results and discussion

### (a) Gender differences

We looked for gender differences using *t*-tests on parametric measures (age, total circles, circles categories, total object suggestions, object categories) and using a *χ*^2^-test on the categorical variable of whether children successfully retrieved the bucket. There was a borderline effect of gender on circles categories: boys drew pictures from a larger number of categories (*M* = 3.00, s.d. = 1.34) than girls (*M* = 2.25, s.d. = 1.02, *t*_38_ = 1.994, *p* = 0.053). There were no other effects (results closest to significance were total circles, *t*_38_ = 1.332, *p* = 0.191 (boys *M* = 3.85, s.d. = 1.53; girls *M* = 3.30, s.d. = 1.03) and total object suggestions, *t*_38_ = 1.333, *p* = 0.191 (boys *M* = 10.70, s.d. = 3.91; girls *M* = 9.20, s.d. = 3.17)).

### (b) Hook-innovation task

Success on the task was defined as making a hook and retrieving the bucket from the tube. Nineteen children (47.5%) succeeded on the task. Two children made a hook on the test trial but failed to succeed in retrieving the bucket from the tube. No child failed to make a hook after the tool manufacture demonstration.

### (c) Divergent thinking tasks

Descriptive statistics for the divergent thinking tasks and analysis of performance are in the electronic supplementary material.

### (d) Individual differences analysis

We conducted a binary logistic regression to predict success or failure on the hook-innovation task. The following measures were entered in to the analysis: Age (in months), gender, total number of different pictures (circles task) and total suggestions score (object uses task). None of these measures was a significant predictor of performance on the hook-innovation task (table 1) and the model appeared to be a poor fit to the data (Cox & Snell *R*^2^ = 0.117). Our model predicted 73% of those who failed the task, but 56% of those who passed.^2^

**Table 1.** Binary logistic regression predicting success on hook-innovation task from divergent thinking measures.

predictor	B	s.e.	Wald	d.f.	*p*-value	odds ratio
age	0.06	0.05	1.47	1	0.229	1.07
gender	−0.81	0.70	1.37	1	0.243	0.44
circles fluency	−0.24	0.27	0.80	1	0.371	0.78
object uses fluency	0.12	0.11	1.17	1	0.279	1.125

There was no evidence that children's innovation success was related to their divergent thinking as measured by the fluency tasks. In Study 2, we turned to a different set of cognitive abilities that we reasoned may predict the development of innovation: executive functions.

## 5. Study 2: executive function

Executive functions are the control processes that are involved in goal-directed actions. As a complex behaviour, it is implausible that tool innovation does not make demands on executive function. Children need to organize their strategies, keep track of what works and switch strategies when necessary. However, we do not know whether differences in executive function are responsible for some children successfully innovating.

Developmentalists tend to think of the executive system as being made up of three dissociated but connected components: inhibitory control, working memory and attentional flexibility [34,35]. Broadly speaking, inhibitory control refers to the ability to stop what one is doing, often characterized as the ability to overcome a prepotent response. Inhibitory control develops markedly between the ages of 3 and 5 years [34], and continues to develop through childhood into adulthood [36]. Working memory is the ability to monitor incoming information and code it according to its relevance for the task at hand. Attentional flexibility refers to the ability to switch between different tasks or operations [37]. In simple terms, task switching requires disengagement with a current but now irrelevant task, and re-engagement with a new relevant task [35]. Children's task switching ability improves dramatically between the ages of 3 and 5 [34], with further advances between the ages of 5 and 11 [38].

As we mentioned earlier, analysis of the tool-innovation task suggests that it must make demands on executive function. When given a tool-innovation problem children must hold in mind the rules of the task and the different components of information. As they engage with the task they must update their knowledge based on feedback and coordinate this knowledge into a useful solution. These activities will tax working memory. fMRI studies with adults have separately indicated that working memory may be important for tool-using actions [38]. Children must use their inhibitory control to suppress irrelevant actions. They must also be able to stop what they are doing if their current strategy is unsuccessful. Finally, children must be able to switch between different strategies. If their current strategy is unsuccessful, children must disengage with the task and re-engage using a new more efficient strategy. Based on this, it seems likely that successful tool innovation in 5- to 8-year-olds may be explained by advances in executive function.

We also considered another way of thinking about executive function that may be relevant to tool innovation: ill-structured problem-solving. The distinction between well-structured and ill-structured problems was first made by Reitman [39]. Reitman defined problems in terms of their start state, goal state and the transformation required to go between the two. If information regarding all three of these components was present, problems were regarded as being well-structured. If information was missing from one or more of the components, Reitman defined the problem as being ill-structured.

Research on ill-structured problems arose from observations of neuropsychological patients. Some patients have been observed to perform at normal levels in tests given to them by experimenters, yet these same patients had difficulty in carrying out every-day simple tasks such as cooking a meal or doing the shopping [40–42]. Based on these peculiar findings, Shallice & Burgess [43] devised new ill-structured tasks that were more closely related to everyday scenarios and required multi-tasking and prospective memory. One task, the Multiple Errands Test, took place in a shopping centre and required patients to retrieve items and information listed for them while following simple rules such as only being able to enter each shop once, and only entering a shop if they purchased something. A laboratory-based version, the Six Elements Test, required patients to complete six tasks of three sub-types while following a set of rules such as not completing two parts of the same sub-type in a row. Shallice and Burgess found that their clinical patients performed comparatively worse on these ill-structured tasks than age- and IQ-matched controls, despite performing at similar levels on traditional executive measures. Researchers concluded that the difficulty of these ill-structured tasks lies in the fact that they require multiple executive functions as described above, yet do not simply reduce to the sum of their parts. Recently, Cutting *et al*. [44] described experimental findings indicating that children had difficulties coordinating information needed to make a novel tool, and interpreted this as suggesting that tool innovation fits the definition of an ill-structured problem. The start and goal states are well defined, but there is information missing about the transformation required to go between the two. In Study 2, we used the Six Parts Test, a measure of ill-structured problem-solving designed for children [45] in the hope of identifying a relationship between innovation and ill-structured problem-solving.

Note that in a pair of recent studies experimental manipulations of one element of executive function, inhibition, were deployed in the hope of improving children's performance [46]. Children were made to delay before attempting to solve the task (delay manipulations have improved children's performance on reasoning tasks that make high inhibitory demands, e.g. [47,48]) and in a separate study they were prompted to switch strategies at regular intervals in case a lack of inhibitory control was leading to perseveration. Neither of these specific inhibitory manipulations improved children's performance. However, we reasoned that it remained important to explore a wider range of executive abilities and also that an individual differences approach may yet yield positive results even if it was not possible to manipulate the inhibitory demands in the tool-innovation task. Overall, we predicted that success on the tool-innovation task would be forecast by performance on executive function measures, especially ill-structured problem-solving.

## 6. Method

### (a) Participants

The final sample consisted of 43 participants aged 6–8 years (25 boys), mean age 7 years 6 months (7;6), range 6;8–8;5, from a primary school in South Birmingham, UK. One child's data were excluded because English was not his first language, reducing the validity of the vocabulary measure we used, and six children did not complete the second testing session.

### (b) Design

Children were tested individually in two sessions by the same experimenter (N.C.). Each session lasted around 15–20 min. The two sessions were administered to children at least 3 days apart and a maximum of 14 days apart. In the first session, participants completed the Six Parts Test from the Behavioural Assessment for Dysexecutive Syndrome for Children (BADS-C) battery [45] and then the British Picture Vocabulary Scale-II (BPVS-II [49]). In the second session, children were given the hook-innovation task and the executive function tasks in a fixed order: Hooks task [8], simple inhibition (‘the pictures task’, adapted from Davidson *et al.* [33]), working memory (a counting recall task based on Alloway *et al*. [50]) and, finally, a task of complex inhibition and task switching (‘the eyes task’, an adaptation of the arrows task from Davidson *et al*. [33]).

### (c) Materials and Procedure

#### (i) Six Parts Test

The Six Parts Test is a subtest from the BADS-C battery [45]. The test comprises three types of task that each has two versions. The green ‘How Many?’ tasks required children to turn over cards to reveal a number of pictures, count the number of pictures and write the total on a piece of paper. The blue ‘What is it?’ tasks required children to turn over cards to reveal pictures, identify the picture and write the name on the paper. All names contained three to five letters; children were aided with spelling if required. The red ‘Sort me’ tasks consisted of two boxes, one containing multiple types of beads, the other containing nuts and bolts. The lids of the boxes contained a picture, and children were required to find the relevant beads or nuts from the boxes that matched the picture and put them in the lids. Children were given instructions as to how to carry out each task and were told they had 5 min to complete as much as they could of each of the six tasks. It was emphasized that they would not be able to complete all of the tasks because they did not have enough time. Additionally, children were given two rules to follow: (i) they must complete a little bit of every single task during the 5 min; (ii) they could not do two types of the same task in a row, e.g. if children were working on the first ‘How many?’ task they could not move on to work on the second ‘How many?’ task next, they must switch to work on one of the ‘What is it?’ or ‘Sort me’ tasks. Children were given 5 min to engage with the test and a timer was in view so that they could check their progress.

*Coding*. Children received an overall score out of 16 for the Six Parts Test, based on the standard scoring strategy: children were awarded two marks for each subtask they attempted (maximum of 12 marks). One mark was deducted for any rule breaks on each of the three types of tasks (up to a maximum of three marks). Marks were added or deducted based on the strategies children used. If children used a clear pattern of responses to avoid breaking the order rule they were awarded two marks, for example, Green 1, Blue 1, Red 1, Green 2, Blue 2, Red 2. If children had a clear strategy for trying to attempt all six parts they were awarded an additional two marks; examples of strategies include undertaking a set number of items on each subtest before switching, or attempting a task for a set amount of time, or a combination of both of these. Children had one mark deducted if they returned to any part three or more times.

#### (ii) British Picture Vocabulary Scale-II

We included a measure of receptive vocabulary alongside our executive battery. Our intention was that we would be able to control for general intelligence to some extent using this as a proxy measure if we found other measures to be predictors.

The BPVS-II [49] is a measure of children's receptive vocabulary. On each trial, children were presented with four outline drawings and were asked to point to the picture that corresponded to a target word spoken by the experimenter. Trials were administered in sets of 12 that increased in difficulty.

Children started with the set that was indicated as being appropriate for their age. The test was terminated if children succeeded on only four or fewer trials within a set. The dependent measure was the total number of correct responses.

#### (iii) Hook-innovation task

Children were presented with the hook-innovation task with slight variations compared to Study 1: we did not include a familiarization phase (Beck *et al*. [8], Experiment 3, found no evidence that the familiarization phase affected performance) and children were presented with a range of materials as in previous versions of the task (e.g. Beck *et al*. [8], Experiment 2). If children failed to retrieve the bucket during the 1 min innovation phase, there followed a two-stage demonstration phase as used by Cutting *et al*. [44]. Children first saw a premade hook. If they then failed to solve the task after a further 30 s the experimenter showed them the manufacturing demonstration. As our interest here is in innovation, not social learning, we do not report on children's performance following the demonstrations. The dependent measure used was whether children solved the task in the first minute.

#### (iv) Executive function tasks^3^

The executive function tasks were presented on a 17 inch screen laptop using E-Prime 2.0 (Psychology Software Tools Inc.). For the ‘Eyes’ and ‘Pictures’ tasks children made responses using two custom-built button boxes. The top faces of the boxes were 12 × 14 cm and they had a depth of 3.5 cm at the back sloping to 2.5 cm at the front. A circular plastic button (diameter 2.5 cm) was present on the top of each box. On the left-hand box, this button was blue and on the right-hand box this button was green. Responses in the counting recall task were made verbally. All tasks had a pseudorandom trial order to ensure that all children had a very similar experience. There were equal numbers of congruent and incongruent trials in the pictures and eyes tasks, and equal numbers of switch and non-switch trials in the eyes task. The pictures and eyes tasks had similar training procedures, where after receiving instructions children received four practice trials with feedback. Children were required to succeed on three out of four practice trials to proceed to the main task. If children did not reach this threshold they received additional sets of four trials until the criterion was reached. The maximum iteration that any child required was two sets.

*Pictures task.* The pictures task is a spatial Simon task that gives a measure of inhibitory control. Children were first presented with two pictures (a monkey and a cat) that were paired with the two response buttons positioned in front of the participant. Children were instructed to press the left-hand (blue) button when they saw the cat and the right-hand (green) button when they saw the monkey. A small picture of each stimulus printed onto card was placed above the relevant response button so as to reduce memory demands. There were 20 trials, in each of which either a cat or a monkey picture was presented in a pseudorandom order on either the left-hand side or right-hand side of the computer screen. Half of the trials required a congruent response, such that the stimulus was presented at the same side as the response button, and half of trials were incongruent, meaning that the picture was presented at the other side to the response button. The incongruent trials were the main source of interest as these allowed measurements of children's ability to inhibit their prepotent response of pressing the response button on the same side as stimulus presentation. Accuracy and response times were recorded by the E-Prime software. Anticipatory responses, less than 200 ms, were removed prior to analyses. Responses greater than 2.5 s.d. from the mean were also removed, as per Davidson *et al*. [33]. A trade-off between accuracy and reaction time was calculated to give an overall processing cost for both the congruent and incongruent trial types. This was calculated by dividing each child's mean reaction time (ms) by the proportion of correct responses such that larger scores represented greater processing costs. The measure of simple inhibitory control used in subsequent correlational analyses was determined by the processing costs for the incongruent trials in comparison to the congruent trials that did not require inhibition.

*Counting recall.* The counting recall task measured children's verbal working memory. On each trial, children were presented with an array of red dots and blue squares and were instructed to count the red dots. Arrays contained between four and seven red dots. The array then disappeared and children were asked to recall verbally how many red dots they had counted. Children began by recalling one screen at a time, and succeeded in this block if they were correct on at least four out of six trials. If they reached this threshold, they proceeded to the next block in which each trial consisted of recalling the numbers of dots in two arrays, then three arrays and so on up to a maximum of five. Each block consisted of six trials, and children needed to achieve four trials correctly to proceed; if children got the first four trials correct they proceeded automatically and were credited as achieving all six correctly. The test was terminated when children were incorrect on three trials within a single block or they had completed all of the available trials. The total number of correct trials was calculated as the dependent measure of working memory. Children received four warm-up trials with feedback prior to starting the task. Two of the warm-up trials had one array and two contained two arrays.

*Eyes task.* The eyes task has both inhibitory and task switching demands. The stimuli in the task were faces presented on the laptop screen. Faces could be presented on either side of the screen and had eyes that looked either straight downwards or diagonally downwards and across at a 45° angle. Children were instructed to press the button the eyes were looking towards. When the eyes looked downwards the correct response was congruent with the side of the screen where the face was presented. When the eyes looked across the screen the response was incongruent with the position the face was presented. To succeed on this task, children must learn two rules: when the eyes are looking downwards they must press the button on the same side as the stimulus, and when the eyes are looking across they must press the button on the opposite side. Burns *et al*. [15] found local switch costs indicating that children treated these as two separate rules and did not combine them in to one simpler rule, i.e. press where the eyes are looking. Children received three blocks of 20 trials; the first block contained all eyes looking downwards, the second block was all eyes looking diagonally, and the third block was a mixture of downwards and diagonal trials.

Three measures were obtained from the eyes task: complex inhibition, local switch cost and global switch cost. The complex inhibition measure was obtained using the same method as the simple inhibition measure outlined in the pictures task, and provided a measure of the processing cost involved in responding to incongruent trials in comparison with congruent trials in the mixed block. In the eyes task, this is termed complex inhibition owing to the increased working memory demands, as unlike in the pictures simple inhibition task there is not a simple mapping between one stimulus and one response. Local switch cost refers to children's ability to switch between different rules within the mixed trial block, that is switching between eyes downwards and eyes downward and across trials. The local switch cost measure is calculated by comparing performance on switch versus non-switch trials within the mixed block. As for the inhibition measures, a processing cost was produced by dividing accuracy on each of the trial types by the proportion of correct trials. The difference between the processing costs for switch and non-switch trials was used as a measure of local switch cost. Global switch cost refers to the cost to children's performance in the mixed block when they know that they might have to switch between rules relative to performance in the congruent or incongruent blocks where no switching is required. Global switch cost was calculated by comparing processing costs for congruent trials that follow congruent trials in the mixed block to congruent trials following congruent trials in the congruent block, and similar for incongruent trials. An average of these two differences was then used as the global switch cost measure.

## 7. Results and discussion

### (a) Gender differences

There were no gender differences on any measures. The results nearest to significance were for global switch, *t*_21.51_ = −1.93, *p* = 0.066 (boys *M* = −348.30, s.d. = 578.53; girls *M* = 308.85, s.d. = 1351.83), simple inhibition, *t*_22.43_ = −1.87, *p* = 0.074 (boys *M* = 386.41, s.d. = 433.64; girls *M* = 824.78, s.d. = 923.44) and age, *t*_41_ = 1.77, *p* = 0.083 (boys *M* = 91.64, s.d. = 6.13; girls *M* = 88.50, s.d. = 5.10).

### (b) Hook-innovation task

On the hook-innovation task, 20 children passed the task without any demonstration. Nineteen children succeeded after seeing the endstate demonstration and the remaining four children passed after having seen the action demonstration.

### (c) Executive function measures

We report descriptive data for all tasks, confirmatory analyses that the executive function tasks were performing as expected, and correlations between measures in the electronic supplementary material.

### (d) Individual differences analysis

We conducted a binary logistic regression to predict success or failure on the hook-innovation task. The following measures were entered in to the analysis: age (in months), gender, receptive vocabulary (BPVS), Six Parts score, simple inhibition (pictures task), working memory (counting recall) and local switch costs (eyes task; table 2). BPVS was the only significant predictor of hook-innovation, 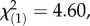 *p* = 0.032, and gender approached significance 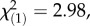 *p* = 0.084. The average raw BPVS score of those children who solved the innovation task was 74.50 (s.d. = 11.86) and of those who did not solve it was 64.61 (s.d. = 10.63). 61% of girls solved the hook-innovation task and 36% of boys. None of the executive function measures made a significant contribution to the regression model and overall the model appeared a poor fit to the data (Cox & Snell *R*^2^ = 0.267). Our model predicted 82.6% of those who failed the task, but only 65% of those who passed.^4^

**Table 2.** Binary logistic regression predicting success on hook-innovation task from executive function measures.

predictor	B	s.e.	Wald	d.f.	*p*-value	odds ratio
age	0.02	0.08	0.08	1	0.777	1.02
gender	1.56	0.90	2.98	1	0.084	4.735
BPVS	0.08	0.04	4.60	1	0.032	1.09
Six Parts score	−0.14	0.10	1.95	1	0.162	0.87
simple inhibition	>0.01	>0.01	0.21	1	0.650	1.00
working memory	0.08	0.16	0.24	1	0.628	1.08
local switch	<0.01	<0.01	<0.01	1	0.988	1.00

Overall, there was no evidence that children's innovation success was related to their executive function. On the other hand, there was a relationship between innovation and receptive vocabulary, which may indicate a role for general intelligence, language ability, or another cognitive process in children's developing innovation.

## 8. General discussion

Around half of 5–8-year-olds solved a hook-innovation task in previous studies: innovation success was remarkably variable in this age group. Neither age nor gender explained this variation in the emergence of tool-making innovation and so we looked to personal characteristics. Considering the demands of the tool-making task, we made two predictions: that innovation may be driven by the ability to generate multiple ideas and that innovation might be reliant on executive function abilities. We examined the first possibility using two divergent thinking tasks, one of which focused on objects. Although performance on the two divergent thinking tasks was related, we could not predict tool-making success based on these scores. In the second study, we used a battery of executive function tasks. This battery included relatively simple executive processes (inhibition, working memory, attentional flexibility) and the more complex ill-structured problem-solving. Once again, there were correlations between executive measures (although not with ill-structured problem-solving), yet none of the executive measures predicted success on the hook-innovation task.

One concern is that our samples of children were relatively small. However, in both studies there were sufficient children to find significant correlations between other measures. Furthermore, there was no hint of any relationship between our innovation measure and the divergent thinking and executive measures we used. Although we are cautious in our interpretation of a lack of relations, our results suggest that a simple explanation for children's difficulty in innovating that implicates divergent thinking or executive function is unlikely to be correct. Studies with larger sample sizes would confirm our preliminary claims, but we also believe that care should be taken before embarking on a large individual differences study of innovation in young children, given the lack of relations with specific personal characteristics that we found in our two studies.

One measure did predict innovation success: children who successfully made a hook in Study 2 scored more highly on the BPVS, a measure of receptive vocabulary. It seems unlikely that the problem-solving aspect of the innovation task makes specific demands on language ability as it is a largely non-verbal task. Yet, one possibility is that children with better comprehension (assumed to be related to other language measures) interpret the instructions or encouragement differently from those with weaker language and thus perform better on the task. However, levels of success are comparable across versions of the task in which children have been given more explicit verbal instructions (to ‘make something’ [21]) and non-verbal demonstrations of manipulating the materials [8] or solving a different task [21]. We suspect that the most probable explanation for this relation is that the BPVS is a proxy here for a general intelligence. Similar arguments have been made by Beck *et al*. [51] and O'Connor *et al*. [52].

One possibility is that the predictor of innovation success is general intelligence itself. Side-stepping the debate of whether such a construct even exists, one reason this does not seem right is that if tool innovation reflects primarily general intelligence then we should expect it to correlate with more of the other measures, at the very least those that also correlated with the BPVS measure: i.e. age and complex inhibition. Yet the possibility that innovation is driven by general intellectual ability is not incompatible with our results. Recently, Muthukrishna *et al*. [53] reported that IQ was negatively related to social learning in human adults, which, if our study did suggest a link between innovation and general intellect, is in line with the possibility that innovation is a complement to social learning (i.e. one is an individual learner or a social learner). However, their further finding that both those with low and high (relative to mid) IQ showed more conformity bias suggested perhaps that high IQ individuals are selective in when they use social information. High IQ individuals may be using both innovation and social learning (see Reader [54] for evidence that innovation and social learning are related). Despite this, the Muthukrishna *et al*. [53] study raises the same question as our own: is it general IQ that supports social learning in their case or innovation in ours, or is the IQ measure (Raven's matrices in Muthukrishna *et al*. [53] and BPVS in our study) reflecting another specific cognitive measure?

The idea that a relationship between innovation and general IQ is in fact masking a more specific relationship would mean that there is another personal characteristic that we have yet to identify. This would be a rather unsatisfactory conclusion to this paper, but perhaps we can suggest some likely candidates. We predicted that divergent thinking and executive functions would relate to the emergence of innovation. Yet, it may be that a different cognitive process is related to innovation (that also relates to general IQ). One possibility is that children's analogical reasoning, their ability to identify similarities between problems and bring relevant information to bear, might be at the heart of innovation [55]. Ratterman & Gentner [56] argue that children's analogical reasoning undergoes a qualitative shift at around 5 years of age, which maps remarkably well on to the changes reported in children's innovation.

Second, it is possible that a personality trait (in the traditional psychological sense) might be involved. Certainly, research with adults has suggested some (e.g. openness) that might be related to innovation, e.g. [13]. Individual differences research in cognitive development has not tended to focus on the role of personality traits to the same extent as it has cognitive abilities (such as executive function) and it is not clear that a personality trait would be closely related to BPVS score or intelligence. The story is likely to be further complicated by the developmental trajectories of such traits, but research suggests that although consistency of personality traits increases across the lifespan (to a peak after age 50) there is at least some consistency in childhood and that there are robust measures of childhood traits [57]. In the future, further individual differences studies should compare performance on innovation to personality traits and other cognitive abilities. But to prevent this becoming a wild goose chase, it will be important to base the choice of measures on our understanding of both innovation and development.

Third, it may be that domain-specific knowledge, rather than a domain general ability is key to understanding the emergence of innovation. Osuirak's theory of human tool use emphasizes the role of technical reasoning, which rests on technical information (‘laws’) being derived from experience with the world [58]. This could lead to the interesting prediction that those children who have more experience interacting with objects should develop the relevant technical knowledge earlier and go on to innovate. There may be potential to manipulate experience with objects experimentally. Another route would be to further explore cross-cultural differences. Nielsen *et al*.'s study [23] showed us that young Bushman children were as unlikely to innovate hooks as urban Australians. However, there may be differences in the rate at which children succeed on this task, depending on the opportunities they have to develop their technical reasoning.

It is important not to overlook a different possibility: that perhaps, in human children at least, personal characteristics do not predict innovative behaviour, indeed, that we are wrong to try to identify some children as innovators. That innovative behaviour is *entirely* random is undermined by both the general developmental evidence that there are gross changes with age, and the specific finding here that BPVS scores predicted innovative behaviour. Perhaps innovation is possible once a certain threshold of intelligence is reached (see Simonton [10] for discussion), yet once this is reached innovation may result from external rather than internal influences. If it is misplaced to seek child innovators, the question remains whether there are adult innovators. It is possible that even if the opportunity for innovation is largely randomly distributed, perhaps those lucky individuals who have a positive experience of it might then be highly motivated to seek out further opportunities, or are encouraged to take risks, or become more open to unconventional possibilities providing good solutions. A developmental approach is thus essential if we are to understand whether innovative ability can be predicted, at least in part, by personal characteristics, or whether the experience of successful, but random, innovation perpetuates itself, regardless of personal characteristics.

It is clear that we do not yet have the answer to why some children solve innovation tasks and others do not. Indeed, we may now also be worried about whether there even are innovators. However, it is clear that an interdisciplinary approach to innovation is needed and understanding the role of development and experience will be critical contributions.

## Supplementary Material

Additional Analyses

## Endnotes

1 Note that the biologists’ use of ‘state’ here describes characteristics that are reasonably stable, unlike the use of the term by psychologists who see states as relatively transient.

2 Because we could not enter multiple measures devised from the same tasks in to the regression analysis, we ran a second analysis using categorical measures of fluency rather than the simple totals: the number of circle categories and the number of object uses categories instead of total number of different pictures and total suggestions score. There was no change to the pattern of results.

3 The executive battery was based on that used by Burns *et al.* [15].

4 We ran a second analysis using global switch costs as a predictor instead of local switch costs. We did not enter both into the same model as they were related measures taken from the same task. There was no change to the pattern of results. A third analysis used complex inhibition as a predictor instead of local switch costs. This measure also came from the same task. There was no change to the pattern of results, although BPVS was now only borderline significant ( *p* = 0.053).

## Data accessibility

Raw data can be obtained from the corresponding author.
