# Supplementary material for: Individual differences in children's innovative problem-solving are not predicted by divergent thinking or executive functions
Source: Philos Trans R Soc Lond B Biol Sci. 2016 Mar 19;371(1690):20150190. doi: 10.1098/rstb.2015.0190 (PMC4780532; doi:10.1098/rstb.2015.0190)
Supplement: Additional Analyses [file rstb20150190supp1.pdf]

Supplementary Material

Individual differences in children's innovative problem solving are not predicted by divergent thinking or executive functions

Sarah R. Beck, Clare Williams, Nicola Cutting, Ian A. Apperly & Jackie Chappell

Study 1

Additional Coding of Object Uses Task

We investigated functional fixedness in our Object Uses task. Following Defeyter et al. (2007), one author (CW) and a blind-coder coded suggestions in terms of whether they were a 'design function', a 'novel function', or 'other suggestion'. A design function is a use of the object which is an extension of its original design. For example, using a brick to "build a house" is a design function. A novel function is a function of the object which is unrelated to its design whilst still plausible given the mechanical properties of the object e.g. size, shape. For example, using a brick "to prop open a door" is a novel function. Other suggestions included suggestions with no end goal e.g. "stand on it", "throw it". A total suggestions score was calculated by summing the design functions, novel functions and other suggestions responses on both the brick and blanket trials.

Children suggested more design functions (M = 4.55) than novel functions (M = 2.12). An ANOVA with age (Younger/Older: spilt at 76 months, the school class boundary) and Function (Design, Novel) showed a main effect of Function, $F(1, 38) = 23.71$, $p < .001$, partial $\eta^2 = 3.84$, but no main effect of ($p = .478$) nor interaction with age ($p = .388$). Descriptive statistics for both the Circles Task and Object Uses Task are shown in Supplementary Table 1.

Supplementary Table 1: Divergent Thinking tasks results.

	Mean (sd)	Minimum Score	Maximum Score
Total Circles	3.57 (1.32)	1	7
Circles Categories	2.63 (1.23)	1	5
Total Object Suggestions	9.95 (3.60)	3	23
Object Categories (functions)	4.15 (1.75)	0	9

Correlation analyses from Study 1

We conducted Spearman's correlations to look for relations between measures. These are reported in Supplementary Table 2.

There were correlations between the Object Categories score and both Circles measures. This suggests that our fluency measures were tapping a more general ability to generate multiple ideas.

Supplementary Table 2: Correlations (Spearman's rho) between measures in Study 1.

	Hook success	Total Circles	Circles Categories	Total Object Suggestions	Object Categories (Functions)
Age	.196	-.064	.072	-.138	.006
Hook success		-.057	.103	.110	.307
Total Circles			removed[a]	.181	.343*
Circles Categories				.226	.422**

Total Object Suggestions					removed[a]

*significant at $p < .05$

**significant at $p < .01$

[a]There were significant correlations ($p < .01$) between the two Circles measures and between the two object task measures. These are removed from the table and further discussion as they are measures calculated from the same data.

Study 2

Executive Function Measures from Study 2

Descriptive statistics are shown in Supplementary Table 3. For the pictures task children were significantly more accurate, t (42) = 7.445, $p < .001$, Cohen's d = 1.14, and faster at responding, t (42) = -6.205, $p < .001$, Cohen's d = 0.95, on the congruent trials than on the incongruent trials. For the mixed block in the eyes task children were more accurate for the congruent trials than they were on the incongruent trials, t (42) = 5.290, $p < .001$, Cohen's d = 0.81, but no significant difference was seen in the reaction times, t (42) = -1.722, $p = .092$. In the eyes mixed block children were more accurate, t (42) = -4.401, $p < .001$, Cohen's d = 0.67, and quicker at responding, t (42) = 6.774, $p < .001$, Cohen's d = 1.03, on the non-switch trials than they were on the switch trials. Children were more accurate, t (42) = 17.824, $p < .001$, Cohen's d = 2.72, and faster, t (42) = -5.027, $p < .001$, Cohen's d = 0.77, in the eyes down (congruent) block than they were in the eyes across (incongruent) block. This finding appears to be driven by surprisingly poor performance for the eyes across block during which many children appeared to persist with the rule they had been using in the eyes downwards block.

Supplementary Table 3: Executive function measures

	Mean Score (sd)	
**Six Parts Task** (max = 16)	7.65 (3.91)	
**Counting and Labelling** (max = 30)	14.09 (2.88)	
	Mean Proportion correct (sd)	Mean Reaction Time (sd) in ms.
**Pictures task**		
Congruent	.90 (.11)	689.08 (139.61)
Incongruent	.67 (.19)	810.358 (140.88)
**Eyes**		
**Mixed Block**		
Congruent	.85 (.15)	900.89 (223.66)
Incongruent	.63 (.26)	956.52 (304.51)
Congruent following congruent	.87(.19)	818.65 (223.00)
Incongruent following incongruent	.72 (.30)	885.84 (299.70)
Switch	.69 (.17)	1016.47 (273.92)
Non-Switch	.78 (.21)	844.55 (215.26)
**Eyes Downwards Block** (matched trials)	.95 (.11)	620.34 (192.76)
**Eyes Across Block** (matched trials)	.40 (.12)	768.09 (272.23)

Correlations analysis from Study 2

We conducted Spearman's correlations to look for relations between measures. These are reported in Supplementary Table 4.

There were correlations between age and i) BPVS, ii) complex inhibition and iii) working memory. BPVS also correlated with complex inhibition. There were also correlations between executive measures: working memory correlated with complex inhibition and also with global switch costs. No measures correlated with the Six Parts task. It is somewhat surprising that this measure did not correlate with age or BPVS, but the lack of correlation with the other executive measures is supportive of claims that ill-structured problem solving is more than the sum of other executive parts.

Supplementary Table 4: correlations between measures in Study 2

	BPVS	Hook Score	Six Parts	Simple Inhibition	Complex Inhibition	Working Memory	Local Switch	Global Switch
Age	.372*	.203	-.051	-.053	-.338*	.397*	.198	-.218
BPVS		.433**	.169	.206	-.401**	.238	.101	-.090
Hook Score			-.093	.169	-.086	.284	.041	.120
Six Parts				-.218	.032	-.067	.061	-.018
Simple Inhibition					.170	-.164	-.014	.281
Complex Inhibition						-.514**	removed[a]	removed[a]

Working Memory	-.009	-.406**
Local Switch		removed[a]

*significant at $p < .05$

**significant at $p < .01$

[a] There was a significant correlation ($p < .01$) between complex inhibition and global switch costs (but not local switch costs). This was removed from the table and further discussion as they are measures calculated from the same data. There was no correlation between local and global switch costs.
